# A Component or Multiple Components of Bleeding Gums May Ameliorate Both Glaucoma and Alzheimer’s Disease

**DOI:** 10.7759/cureus.21004

**Published:** 2022-01-07

**Authors:** Steven Lehrer, Peter H Rheinstein, James Schmeidler

**Affiliations:** 1 Radiation Oncology, Icahn School of Medicine at Mount Sinai, New York, USA; 2 Family Medicine, Severn Health Solutions, Severna Park, USA; 3 Psychiatry, Icahn School of Medicine at Mount Sinai, New York, USA

**Keywords:** prostaglandins, periodontitis, alzheimer’s disease, neurodegeneration, glaucoma

## Abstract

Background: Although clinical studies have shown an increased prevalence of primary open-angle glaucoma (POAG) in patients with Alzheimer’s disease (AD), a population-based epidemiologic study from Denmark found no increased risk of Alzheimer’s disease in patients with glaucoma, and other studies have failed to demonstrate a link. However, a possible relationship between POAG and AD might manifest in their association with oral pathology. Dental caries, periodontal disease, stomatitis, and the related inflammatory burden increase AD risk, while oral pathology and the oral microbiome correlate with POAG vulnerability. To further examine the relationship, we analyzed POAG, AD, and oral disease in the UK Biobank (UKBB) cohort.

Methods: Our analysis included all subjects with POAG and AD. POAG diagnosis was ascertained using the 10th Revision of the International Classification of Diseases (ICD-10), H40.11. AD diagnosis was ascertained using the 10th Revision of the International Classification of Diseases (ICD-10), G30. Oral cavity, ulceration, stomatitis, periodontitis, teeth, and dental problems were in UKBB data field 6149.

Results: A “yes” answer to a question about bleeding gums is associated with a greater proportional POAG reduction (24.2%) than a “yes” answer to having none of the six listed problems (6.3%). Similarly, bleeding gums were associated with a greater proportional AD reduction (46.2% versus 16.9%). Logistic regression controlling for age and sex showed that bleeding gums (no/yes) were negatively associated with AD (odds ratio (OR) = 0.713, 95% confidence interval (CI) = 0.521-0.976, p = 0.035). Age-weighted least-squares linear regression showed that the lower corneal-compensated intraocular pressure (IOP) in the left eye was associated with bleeding gums (unstandardized regression coefficient = -0.174, p < 0.001), controlling for type 2 diabetes and past smoking.

Conclusion: It is difficult to predict what component or components of periodontal inflammation might be ameliorating POAG and AD. Prostaglandin is a possibility. Identification of the component or components could lead to new treatments for POAG and AD. Further studies are warranted.

## Introduction

Glaucoma is a collection of ocular neuropathies marked by the degeneration of retinal ganglion cells (RGCs) and their axons, which results in a distinctive optic nerve appearance and visual field abnormalities. The most prevalent type of glaucoma is primary open-angle glaucoma (POAG). Glaucoma can lead to vision impairment and eventually blindness if not treated. Currently, intraocular pressure (IOP) is the sole known therapeutic component.

The most prevalent cause of dementia in the elderly is Alzheimer’s disease (AD), a neurodegenerative condition that develops over time and is defined clinically as growing cognitive and behavioral impairment. β-Amyloid plaques and neurofibrillary tangles are the pathological hallmarks of Alzheimer’s disease. The epsilon 4 allele of apolipoprotein E (APOE), which has been linked to POAG [1], is a substantial genetic risk factor for late-onset AD. However, the evidence on whether APOE polymorphisms enhance the incidence of POAG is mixed [2].

Structural examinations of optic nerves from patients with AD have revealed degeneration and loss of retinal ganglion cells, such as POAG. Caspase activation and the aberrant processing of amyloid precursor protein (APP), both essential processes in Alzheimer’s disease, have been demonstrated in a rat model of chronic ocular hypertension. In the same type of experimental glaucoma, β-amyloid has been linked to retinal ganglion cell (RGC) apoptosis. Therefore, POAG and AD may be part of the same age-related neurodegenerative process [3].

Although clinical studies have shown an increased prevalence of POAG in patients with Alzheimer’s disease [2], a population-based epidemiologic study from Denmark found no increased risk of Alzheimer’s disease in patients with glaucoma [4]. Other studies have also failed to demonstrate a link [5-7].

A possible relationship between POAG and AD might manifest in their association with oral pathology. Dental caries, periodontal disease, stomatitis, and the related inflammatory burden increase AD risk [8,9], while oral pathology and the oral microbiome correlate with POAG vulnerability [10-13].

To further examine the relationship, we analyzed POAG, AD, and oral disease in the UK Biobank (UKBB) cohort.

## Materials and methods

The UK Biobank is a major men’s and women’s prospective observational study. Between 2006 and 2010, participants were recruited from 22 different centers across England, Wales, and Scotland, and they are still being followed for future health events [14]. The UKBB uses the same technique as the continuing Framingham Heart Study, with the exception that UKBB gathers postmortem samples, which Framingham does not [15].

Our UK Biobank application was approved as UKB project 57245 (SL and PHR). Our analysis included all subjects with POAG and AD, including concomitant. POAG diagnosis was ascertained using the 10th Revision of the International Classification of Diseases (ICD-10), H40.11. AD diagnosis was ascertained using the 10th Revision of the International Classification of Diseases (ICD-10), G30. Oral and dental problems were in UKBB data field 6149. A touchscreen question was presented to all subjects: “Do you have any of the following? (You can select more than one answer).” The choices were mouth ulcers, painful gums, bleeding gums, loose teeth, toothache, dentures, or none of the above. A subject could enter up to six responses. Data were collected during a single session, and no follow-up or longitudinal data were collected.

Data processing was performed on Minerva, a Linux mainframe with Centos 7.6, at the Icahn School of Medicine at Mount Sinai. We used the UK Biobank Data Parser (ukbb parser), a python-based package that allows easy interfacing with the large UK Biobank dataset [16]. Statistical analysis (Fisher’s exact test and logistic regression) was done using SPSS 25 and R.

## Results

The mean age of 349,549 subjects was 56 ± 8 (mean ± SD), 54% were female and 46% were male, and 95% were White British. Additional demographics are in the following tables.

Oral cavity problems in subjects versus POAG (no/yes) are in Table 1. Variability is significant (p < 0.001, chi-square test). A “yes” answer to a question about bleeding gums is associated with a greater proportional POAG reduction (24.2%) than a “no” answer (6.3%).

**Table 1:** Oral cavity problems in subjects versus primary open-angle glaucoma (POAG) (no/yes). Variability is significant (p < 0.001, chi-square test). Note that the percentage of “yes” answers with bleeding gums and POAG (10%) was lower than the percentage of “yes” answers with bleeding gums and no POAG (13.2%). Bleeding gums appear to be associated with an even greater proportional reduction (PR) (10%/13.2% = 0.758; 1 - 0.758 = 0.242 = 24.2%) than “none” (56.7%/60.5% = 0.937; 1 - 0.937 = 0.063 = 6.3%). A negative PR represents a proportional increase. In Tables 1 and 3, the total at the bottom of the table is equal to the sum of the counts for the conditions. Since the participants could select more than one condition, the total at the bottom of a column is not a total number of participants (so that the percentage for bleeding gums would be the percentage of the participants in that column with bleeding gums) but the number of “yes” answers (so the percentage for bleeding gums would be the percentage of “yes” answers in that column with bleeding gums). In Table 2, each participant can only appear once in a column.

Mouth problems		POAG	POAG	Total	PR
		No	Yes		
None	Count	289,973	1,815	291,788	
	% within POAG	60.5%	56.7%	60.5%	6.3%
Mouth ulcers	Count	48,277	286	48,563	
	% within POAG	10.1%	8.9%	10.5%	15.2%
Painful gums	Count	15,053	102	15,155	
	% within POAG	3.1%	3.2%	3.1%	-3.2%
Bleeding gums	Count	63,422	321	63,743	
	% within POAG	13.2%	10%	13.2%	24.2%
Loose teeth	Count	20,703	149	20,852	
	% within POAG	4.3%	4.7%	4.3%	-9.3%
Toothache	Count	19,473	140	19,613	
	% within POAG	4.1%	4.4%	4.1%	-7.3%
Dentures	Count	80,982	795	81,777	
	% within POAG	16.9%	24.8%	17%	-45.9%
Total	Count	47,9016	3,203	482,219	
	% within POAG	100%	100%	100%	

The cross-tabulation, bleeding gums versus POAG, is in Table 2. Of the subjects without bleeding gums, 0.60% had POAG, and 0.50% of subjects with bleeding gums had POAG (p < 0.001, two-tailed Fisher’s exact test). The effect of mouth ulcers on POAG was insignificant (p = 0.323, two-sided Fisher’s exact test). The effect of painful gums on POAG was insignificant (p = 0.125, two-sided Fisher’s exact test).

**Table 2:** Cross-tabulation, bleeding gums versus primary open-angle glaucoma (POAG). Of the subjects without bleeding gums, 0.6% had POAG, and 0.5% of subjects with bleeding gums had POAG (p = 0.001, two-tailed Fisher’s exact test.

	No POAG	POAG	Total
No bleeding gums	304,672	1,890	306,562
%	99.4%	0.6%	100%
Bleeding gums	63,422	321	63,743
%	99.5%	0.5%	100%
Total	368,094	2,211	370,305
%	99.4%	0.6%	100%

The cross-tabulation, loose teeth versus POAG, showed that 0.6% of the subjects without loose teeth had POAG and that 0.8% of the subjects with loose teeth had POAG (p < 0.001, two-tailed Fisher’s exact test). The cross-tabulation, dentures versus POAG, also showed that 0.60% of the subjects without dentures had POAG and that 1% of the subjects with dentures had POAG (p < 0.001, two-tailed Fisher’s exact test). The effect of toothache on POAG was insignificant (p = 0.072, two-sided Fisher’s exact test). The relationship of tooth problems to POAG has been reported previously [13].

The corneal-compensated IOP of the left eye in 67,152 subjects without bleeding gums was 16.11 ± 4.28 and in 12,469 subjects with bleeding gums was 15.95 ± 4.41 (p < 0.001, two-tailed t-test). Age-weighted least-squares linear regression was performed. The corneal-compensated IOP of the left eye was the dependent variable, and bleeding gums (no/yes), past smoking (no/yes), and type 2 diabetes (no/yes) were the independent variables. Bleeding gums were significantly correlated with lower IOP (unstandardized regression coefficient B = -0.174, p < 0.001) and independent of the effects of type 2 diabetes (unstandardized regression coefficient B = 0.413, p < 0.001) and past smoking (unstandardized regression coefficient B = 0.066, p = 0.037).

Table 3 shows mouth problems in subjects versus AD (no/yes). Variability is significant (p < 0.001, chi-square test). A “yes” answer to a question about bleeding gums is associated with a greater proportional AD reduction (46.2%) than a “no” answer (16.9%). 

**Table 3:** Mouth problems in subjects versus Alzheimer’s disease (no/yes). Variability is significant (p < 0.001, chi-square test). Note that the percentage of “yes” answers with bleeding gums and AD (7.1%) was lower than the percentage of “yes” answers with bleeding gums and no AD (13.2%). Bleeding gums appear to be associated with an even greater AD proportional reduction (PR) (46.2%) than “none” (16.9%). Dentures are associated with a 101.2% proportional increase of AD.

Mouth problems		AD	AD	Total	PR
		No	Yes		
None	Count	291,325	463	291,788	
	% within AD	60.50%	50.30%	60.50%	16.9%
Mouth ulcers	Count	48,471	92	48,563	
	% within AD	10.10%	10%	10.10%	1%
Painful gums	Count	15,115	40	15,155	
	% within AD	3.10%	4.30%	3.10%	-38.7%
Bleeding gums	Count	63,678	65	63,743	
	% within AD	13.20%	7.10%	13.20%	46.2%
Loose teeth	Count	20,815	37	20,852	
	% within AD	4.30%	4%	4.30%	7%
Toothache	Count	19,580	33	19,613	
	% within AD	4.10%	3.60%	4.20%	14.3%
Dentures	Count	81,462	315	81,777	
	% within AD	16.90%	34.20%	17%	-101.2%
Total	Count	481,299	920	482,219	

The cross-tabulation, bleeding gums versus AD, is in Table 4. Of the subjects without bleeding gums, 0.20% had AD, and 0.10% of the subjects with bleeding gums had AD (p < 0.001, two-tailed Fisher’s exact test). The cross-tabulation, dentures versus AD, also showed that 0.2% of the subjects without dentures had AD and that 0.4% of subjects with dentures had AD (p < 0.001, two-tailed Fisher’s exact test). Subjects with dentures have a higher risk of AD [17]. The effect of mouth ulcers on AD was insignificant (p = 0.115, two-sided Fisher’s exact test). The effect of painful gums on AD was insignificant (p = 0.096, two-sided Fisher’s exact test). The effect of loose teeth on AD was insignificant (p = 0.739, two-sided Fisher’s exact test). The effect of toothache on AD was insignificant (p = 0.815, two-sided Fisher’s exact test).

**Table 4:** Cross-tabulation, bleeding gums versus Alzheimer’s disease (AD). Of the subjects without bleeding gums, 0.20% had AD, and 0.10% of the subjects with bleeding gums had AD (p = 0.002, two-tailed Fisher’s exact test).

Bleeding gums	No AD	AD	Total
No	306,094	468	306,562
%	99.8%	0.2%	100%
Yes	63,678	65	63,743
%	99.9%	0.1%	100%
Total	369,772	533	370,305
%	99.9%	0.1%	100%

Logistic regression and 95% confidence interval (CI) lower bound (LB) and upper bound (UB) of AD (no/yes) (dependent variable) and bleeding gums (no/yes), age, and sex (independent variables) are shown in Table 5. Bleeding gums were associated with AD, with an OR of 0.713 (95% CI = 0.521-0.976). Every year of age was associated with AD, with an OR of 1.210 (95% CI = 1.189-1.232). Male sex was associated with AD, with an OR of 1.274 (95% CI = 1.070-1.517). A previous study showed that sex was associated with the risk of dementia in the UK Biobank cohort, and the risk was lower in women than in men [18].

**Table 5:** Logistic regression and 95% confidence interval lower bound (LB) and upper bound (UB) of Alzheimer’s disease (no/yes) (dependent variable) and bleeding gums (no/yes), age, and sex (independent variables). Bleeding gums were associated with AD, with an OR of 0.713. Every year of age was associated with AD, with an OR of 1.210. Male sex was associated with AD, with an OR of 1.274.

	95% LB	OR	95% UB	p value
Bleeding gums	0.521	0.713	0.976	0.035
Age	1.189	1.210	1.232	<0.001
Sex	1.070	1.274	1.517	0.007

There was no direct relationship between POAG and AD (p = 0.155, two-tailed Fisher’s exact test). Other studies have reported the same lack of relationship, as noted above [4-7].

## Discussion

A “yes” answer to a question about bleeding gums is associated with a greater proportional POAG reduction (24.2%) than a “no” answer (6.3%). The corneal-compensated left eye IOP difference in subjects with versus without bleeding gums was statistically significant, but the effect size was quite small. While clinically insignificant, the small effect size may indicate that bleeding gums are elaborating only a small amount of the substance or substances favorably affecting POAG and AD. If these substances could be identified and larger amounts administered, the effect size could be clinically significant.

The protective effect of bleeding gums against POAG and AD is probably not due to bacteria. The major periodontal pathogens *Porphyromonas gingivalis*, a keystone pathobiont in periodontitis, and *Bacteroides forsythus* and their bacterial components have been found in the brains of patients with AD and seem to provoke the inflammation that is a feature of AD [9]. There is growing interest in the role of the gingipains produced by *Porphyromonas gingivalis* on the enhancement of amyloid production and possible tangling of Tau proteins in Alzheimer’s disease [19,20].

POAG pathophysiology is influenced by the oral microbiome. Experiments in animal models suggest that higher bacterial loads can cause neurodegeneration by activating microglia in the retina and optic nerve, mediated by toll-like receptor 4 (TLR4) signaling and complement overexpression [10].

Cytokines, such as interleukin-1 and tumor necrosis factor, and chemokines are produced during periodontal inflammation [21]. However, these substances raise IOP rather than lower it [22], and the production of TNF-α during periodontitis blocks insulin receptors and may contribute to an adverse effect on the control of type 2 diabetes [23].

Prostaglandins are a key component of periodontitis and stimulate bone destruction by activating matrix metalloproteinases [24]. However, prostaglandins reduce IOP, and prostaglandin analogs are available for the topical treatment of POAG [25]. Prostaglandin E2 may protect against advanced AD [26], although prostaglandins can act as neurotoxins and may be a cause of AD [27,28].

A limitation in our analysis includes having different variables in Table 1 versus Table 2 and Table 3 versus Table 4. The retrospective analysis of UK Biobank data lends itself to this arrangement. Moreover, the reduced significance in Table 5 for bleeding gums compared with Table 3 and Table 4 suggests the significant contribution of the Table 5 covariates. The retrospective UKBB data are valuable for hypothesis generation, but a prospective study is needed.

Having none of the listed mouth conditions is associated with a lower incidence of POAG; in other words, mouth conditions, in general, predispose to POAG. Mouth ulcers are associated with a lower incidence of POAG, suggesting that mouth ulcers may elaborate the same POAG/AD protective substance or substances as bleeding gums. Alternately, the lower incidence of POAG in subjects with bleeding gums may result from some of these individuals ingesting something such as aspirin, which is known to cause bleeding gums [29] and is sometimes posited as a treatment for glaucoma [30].

## Conclusions

It is difficult to predict what component or components of bleeding gums or periodontal inflammation might be ameliorating POAG and AD, although prostaglandins are prime candidates. Conclusive identification of the component or components could lead to new treatments for POAG and AD. Prostaglandins are already known to be beneficial for POAG, but their effect on AD is not well established. New treatments for AD are sorely needed, and prostaglandins might be one. Further studies are warranted.
